# Automated Assessment of Retinal Vascular Integrity Across Species

**DOI:** 10.1167/iovs.66.15.46

**Published:** 2025-12-15

**Authors:** Jeffrey O'Callaghan, Natalie Hudson, Nicole Hanley, Avril Reddy, Aisling Naylor, Alan Hopkins, Matthew O'Riordan, Fionn O'Leary, Deirdre Harford, Rory Holohan, Denis Nevrov, Eleanor Demmons, Matthew S. Lawrence, Sarah L. Doyle, Peter D. Westenskow, Mark Cahill, Matthew Campbell

**Affiliations:** 1 Smurfit Institute of Genetics, Trinity College Dublin, Dublin, Ireland; 2 Royal Victoria Eye and Ear Hospital, Research Foundation, Dublin, Ireland; 3 Progressive Vision Research, Dublin, Ireland; 4 Virscio, Inc., New Haven, Connecticut, United States; 5 Trinity College Institute of Neuroscience, Trinity College Dublin, Dublin, Ireland; 6 Department of Clinical Medicine, School of Medicine, Trinity College Dublin, Dublin, Ireland; 7 Pharma Research and Early Development, F. Hoffmann–La Roche, Basel, Switzerland

**Keywords:** fluorescein angiography, blood retina barrier, circadian rhythm, retinal vasculature

## Abstract

**Purpose:**

To develop an automated approach for the analysis of fundus fluorescein angiography (FFA) images and apply this methodology to examine inner blood–retina barrier (iBRB) circadian integrity across species and age.

**Methods:**

We acquired and quantified retinal images from mouse, non-human primate (NHP), and human eyes taken at the morning and in the evening. To facilitate the analysis, we developed and validated the Fluorescent Ocular Vasculature Analysis Suite (FOVAS), allowing for the quantification of large temporal retinal FFA datasets, with automated analysis, graphing, and reporting. We then compared fluorescein intensity at various microvasculature regions and ages and examined fluorescein signal at distinct phases along a 10-minute imaging session.

**Results:**

We detected a significant circadian-related difference in iBRB leakage between the morning and the evening in young adult mice, NHPs, and humans, using the validated FOVAS to quantify the FFA images. This difference was reduced with age across species.

**Conclusions:**

FOVAS successfully quantified FFA images demonstrating high sensitivity for the detection of changes in vascular integrity across species and time.

Fundus fluorescein angiography (FFA) is typically used to diagnose retinal or choroidal vascular diseases, monitor disease progression, assess treatment efficacy, and study retinal vascular features.^1^^,^^2^ By examining fluorescence patterns, pathological blood flow or vessel permeability can be inferred and can serve as a robust indicator of diseases such as age-related macular degeneration, diabetic retinopathy, diabetic macular edema, ocular tumors, and other optic neuropathies. Although FFA has broad application, its interpretation requires an experienced ophthalmologist, and agreement can vary considerably between observers.^3^ Additionally, subtle subclinical changes in microvascular integrity may also be overlooked, as they may not be obvious on first observation. Multiple lines of evidence now suggest that changes to the integrity of the blood–brain barrier (BBB) in the aging brain can prime and exacerbate damage to delicate neural tissues.^4^^–^^6^ Increased permeability of the BBB has also been recognized as important in serving as an indicator of disease progression.^7^ In this regard, it must be noted that the human retina is simply an extension of the central nervous system (CNS), being the only portion of the CNS that is not encased in bone. Therefore, the retina is an extension of the brain, and the microvessels of the retina share properties almost identical to the BBB and are referred to as the inner blood–retina barrier (iBRB).^8^ It is important to note that this is anatomically distinct from the outer blood–retina barrier (oBRB) which refers to the barrier properties of the retinal pigment epithelium).^9^

Given the small, sometimes microscopic, scale at which changes to the iBRB may occur and be initiated, there are currently limitations to what ophthalmologists may identify during a standard clinical examination. Therefore, it is clear that there is currently a need for a software platform with utility across instruments and species that will allow for objective quantification of iBRB integrity. In recent years, several advances have been made by various groups in quantifying retinal vascular integrity during FFA, including vessel segmentation, leakage, and blood flow rate^10^^–^^16^; however, these methods are not commercially available for use by clinical teams or those without a background in coding, and wide implementation of FFA quantification remains limited as a diagnostic tool. Added to this, the use of animal models of retinal disease is critical for the identification of novel therapeutic targets and screening of therapeutic efficacy of investigational new drugs. In that regard, methods aimed at synergizing the tools used in preclinical and clinical data processing are critical to ensure translational success.

In order to be able to further investigate the workings of the iBRB, we developed the Fluorescent Ocular Vasculature Analysis Suite (FOVAS) as an easy-to-use and accessible cross species platform for automated quantification of FFA image datasets, with a simple graphical user interface, automated image registration, and analysis of regional and temporal fluorescein signal intensity. In doing so, we were able to identify a distinct difference in fluorescein signal in the morning compared to the evening in multiple species, including humans. Intriguingly, we discovered that this difference in iBRB integrity appears to be fundamentally lost with age.

## Materials and Methods

In this study, we determine the circadian nature of the vascular permeability of mouse, non-human primate (NHP), and human eyes. Circadian differences observed in the retinal vasculature are the perfect candidate to demonstrate the features of FOVAS, as such differences may not be readily detectable by the naked eye, and manual image processing and analysis using standard means are often extremely time consuming.

### Image Acquisition

For the collection of new data, it was important that the same image acquisition settings were used within each study to minimize technical variability in the fluorescein signals. To this effect, exposure settings were tested across a number of test subjects before gain settings were chosen for each instrument–species pairing. When a suitable gain was chosen, this was kept consistent for all participants of that experiment (image brightness control = manual, sensitivity = 50–55). The automatic real time (ART) mean function was enabled for image capture to improve the signal to noise (ART = 50–80). Additionally, fluorescein concentrations were kept constant within experiments, as described in the relevant sections below. Image export settings were also kept consistent for each experiment to further reduce any signal intensity variability arising out of the raw images. This included file type, image resolution, and image dimensions. Single images within the series for each subject were assessed by two investigators (JOC and NH) in a first-pass filtering step to exclude images of very poor quality including low brightness or focus. The treatment of animals and procedures used in this study were carried out in accordance with regulations set out by the Health Products Regulatory Authority (HPRA), responsible for the correct implementation of EU directive 2010/63/EU, and the studies were conducted in line with the ARVO Statement for the Use of Animals in Ophthalmic and Vision Research.

#### Mouse Fundus Fluorescein Angiography

C57BL/6J mice were sourced from The Jackson Laboratory (Bar Harbor, ME, USA) and bred onsite at the Smurfit Institute of Genetics. All mice were kept on a 12-hour light/12-hour dark cycle with temperature and humidity maintained at 18° to 23°C and 40% to 50%, respectively. Mice were provided ad libitum access to standard food and water. FFA was performed using a Heidelberg SPECTRALIS (Heidelberg Engineering, Heidelberg, Germany). Pupils were dilated with 1% tropicamide and 2.5% phenylephrine eye drops, and mice were anesthetized by a mixture of ketamine (100 mg/kg) and medetomidine (0.25 mg/kg). For FFA, mice were intraperitoneally injected with sodium fluorescein (2mg/mL) at the same volume/weight (100 µL/20*g*) to visualize blood vessels. FFA images were captured with a 30° angle view from 30 seconds to 10 minutes every 30 seconds. Anesthesia was reversed with atipamezole hydrochloride (1 mg/kg). Imaging was performed at 8:00 AM and 8:00 PM for the morning and evening time points, respectively.

#### NHP Fundus Fluorescein Angiography

NHP studies were conducted in a Virscio laboratory at the St. Kitts Biomedical Research Foundation (St. Kitts, West Indies) in a protocol^17^ approved by the Institutional Animal Care and Use Committee is a federal body. FFA images from six adult African green monkeys (*Chlorocebus sabaeus*) were used in this study. Fundus color photography and FFA were conducted in six animals anesthetized with ketamine and xylazine. Imaging was performed using a retinal camera (TRC-50X; Topcon America Corporation, Oakland, NJ, USA) equipped with Canon D4 digital hardware (Tokyo, Japan) and analyzed with a NewVision Fundus image analysis system (NewVision Fundus, San Francisco, CA, USA). To prevent residual fluorescein interference, angiography of the left eye was carried out 6 hours after imaging of the right eye, allowing sufficient time for fluorescein washout between sessions. Prior to fluorescein injection, color and red-free fundus images were obtained from both eyes. FFA was initiated by intravenous administration of 10% sodium fluorescein (0.1 mL/kg) via the saphenous vein. Serial angiographic images were then captured using a 50° field centered on the posterior pole, beginning immediately after injection and continuing throughout the first minute, followed by additional captures at 2, 3, and 6 minutes. To confirm inter-eye consistency in fluorescein distribution and vascular appearance, a single angiogram of the contralateral eye was obtained immediately after the 6-minute time point. This quality control check was qualified by Virscio prior to analysis of the entire dataset. Imaging was performed at 6:00 AM and 6:00 PM for the morning and evening time points, respectively.

#### Human Fundus Fluorescein Angiography

Ethical approval for this study was obtained from the Royal Victoria Eye and Ear Hospital Research Foundation before study initiation. Volunteers were recruited from Dublin, Ireland, and informed consent was obtained from all participants prior to involvement. Visual acuity and intraocular pressure measurements were performed prior to imaging. Fundus color photography was performed using a retinal camera (Topcon TRC-50X) with Canon D4 digital imaging hardware, and FFA analysis was performed using the Heidelberg SPECTRALIS. Pupils were dilated with 1% tropicamide. Sodium fluorescein (500 µg), followed by a 5-mL flush of 0.9% sodium chloride, was injected via a peripheral cannulation site to visualize the blood vessels of the posterior pole. FFA images were captured from 15 seconds onward up to 10 minutes. The images were captured with a 30° angle view, and hardware normalization was disabled. Between the morning and evening visits, a period of at least 24 hours was given to ensure flush out of any residual sodium fluorescein. HEYEX 1.7.1.0 was used to capture images. Fundus images were independently reviewed by a consultant ophthalmologist for grading. Fifteen participants of this younger cohort have been previously described,^17^ which we have now increased to a total of 32 for this study. A total of 17 participants were recruited for the older adult cohort. Participants were imaged at approximately 8:00 AM (7:00–9:00 AM) and 8:00 PM (6:30–8:00 PM) for the morning and evening time points respectively. The key demographics for all of our cohorts are described in the Table.

**Table.** Key Demographics for Subjects Across the Human and Animal Studies

Variable	Young Adult Human Cohort	Older Adult Human Cohort	NHP	Young Adult Mouse	Older Adult Mouse
Number	33	17	5	15	38
Sex (M/F), *n*	24/9	10/7	2/3	9/6	16/22
Age, average	25 y	72 y	5 y	7 mo	19 mo
Age range	20–30 y	64–85 y	4–6 y	3–12 mo	18–24 mo
Eyes included in analysis per subject	OS	Eye with best vision^*^	OS + OD	OS	OS

The number of participants is shown along with sex and age differences. The eyes included in the analyses are also denoted for clarity.

* The eye with better vision was determined by a clinician after fundus images were taken. Eyes with clear fundus were preferred over those with cataracts or other abnormalities only present in one eye.

### FOVAS Application

As mentioned in the introduction, we developed FOVAS to facilitate the study of the iBRB. This FOVAS application was written in MATLAB R2023b (MathWorks, Natick, MA, USA). App functionality prioritized the batch processing of multiple images from multiple participants across studies, as well as the automated generation of statistical results. User workflow is spread among three categories described below.

#### File Conversion

Image inputs are first converted into FOVAS-readable formats by ingesting via a flexible decoder that accepts multiple medical and open-source file formats. The acquisition time of each image after fluorescein injection is also determined by parsing embedded metadata or, when absent, by automated text detection. Images are then automatically sorted into a study folder for downstream processing.

#### Image Processing

When user-defined parameters have been selected, the image processing stage can be initiated. Image preprocessing involves any necessary cropping to remove machine info panels adjacent to the image, non-uniform background illumination correction using a morphology-based high-pass filter to correct for non-uniform textures such as shadows on the image, resizing large images to save on memory, and any other required acquisition hardware-specific preprocessing. Images are then registered, aligning all images to facilitate temporal analysis. This is done through computer vision methods by means of a hybrid feature-based approach, which may invoke Orientated FAST and Rotated BRIEF (ORB)-style descriptors^18^ or frequency-domain correlation,^19^ depending on the species from which the images originated. The registration routine is automatically tuned to the animal model: a keypoint‑based method for primates and a frequency‑domain method for rodents, which was found to be more robust for murine images. Murine images tend to not result in a large enough number of keypoints for robust registration. Keypoints are automatically generated by the Features from Accelerated Segment Test (FAST) algorithm up to a maximum of 15,000 per image. Frames are automatically excluded if they do not register correctly or fail to meet registration criteria. For example, a dark image would generate too few keypoints to create a successful transformation and would be flagged for exclusion. When registered, the maximum intensity projection (MIP) is presented to the user in order to define the position of the macula. This is used as the center point for the Early Treatment Diabetic Retinopathy Study (ETDRS) grid.^20^ This process anatomically segments the macular region into fovea, perifovea, parafovea, and extrafoveal regions of interest. Species-specific vessel masking is applied before intensity extraction. The fluorescence profile within each region of interest is quantified by computing the pixel intensity. Throughout this process, quality control images are generated, including montages, stacks, and copies of the MIP for optional manual validation.

#### Image Analysis

For analysis, different methods can be selected/deselected on the interface; however, the default pipeline involves testing for normality using an Anderson–Darling test (to automatically define downstream statistical methods) and normalizing data if required. All studies in this manuscript generated non-normal distributions for the processed image intensity data, thus analysis methods were chosen accordingly. Fluorescein signal intensity versus time was plotted for each repeated measure for every participant. Cumulative intensity was then plotted, calculated from data only from time points present in all participants. The software produced separate plots that summarize the fluorescence signal over user‑defined temporal segments, typically aligned with the conventional early (first minute), intermediate (2–4 minutes), and late (>4 minutes) angiographic phases. In the present study, time ranges for the mid- and late angiographic phases were defined by the user on the software to examine the temporal nature of the fluorescein signal. Signal decay was also analyzed, looking at the rate of fluorescein clearance which was, for a given time point, taken as the percentage of the max intensity of the temporal profile, usually in the early phase. Users may also implement user-guided frame selection and vessel masking to enhance microvasculature representation, thereby improving the accuracy of registration and segmentation workflows.

#### Statistical Analysis

Statistical analysis is automatically performed on each of the data tables generated for each analysis method. As part of the statistical pipeline, data are inspected to test if they follow a normal distribution according to an Anderson–Darling normality test. The results determine downstream statistical methods and central tendency measures. Here, paired or unpaired *t*-tests, ANOVA or mixed-effects models, and repeated-measures sample testing methods were applied where appropriate. Data and statistical results are then exported for user interpretation. Specific details on statistical tests for each dataset used are highlighted in the Results section.

#### Validation

FOVAS was initially validated using a previously reported NHP dataset^17^ that had been manually analyzed with ImageJ (National Institutes of Health, Bethesda, MD, USA). Applying FOVAS to the same data yielded leakage measurements consistent with those reported in the original study, demonstrating agreement between our automated pipeline and established manual quantification. Following this validation, FOVAS was used to analyze iBRB integrity in mice, NHPs, and humans. The FOVAS software, developed in MATLAB, incorporates proprietary algorithms currently under commercial protection. For this reason, the source code and executables cannot be shared publicly at this time.

## Results

### FOVAS Facilitates Quantitative FFA Across Species

**Figure 1.**
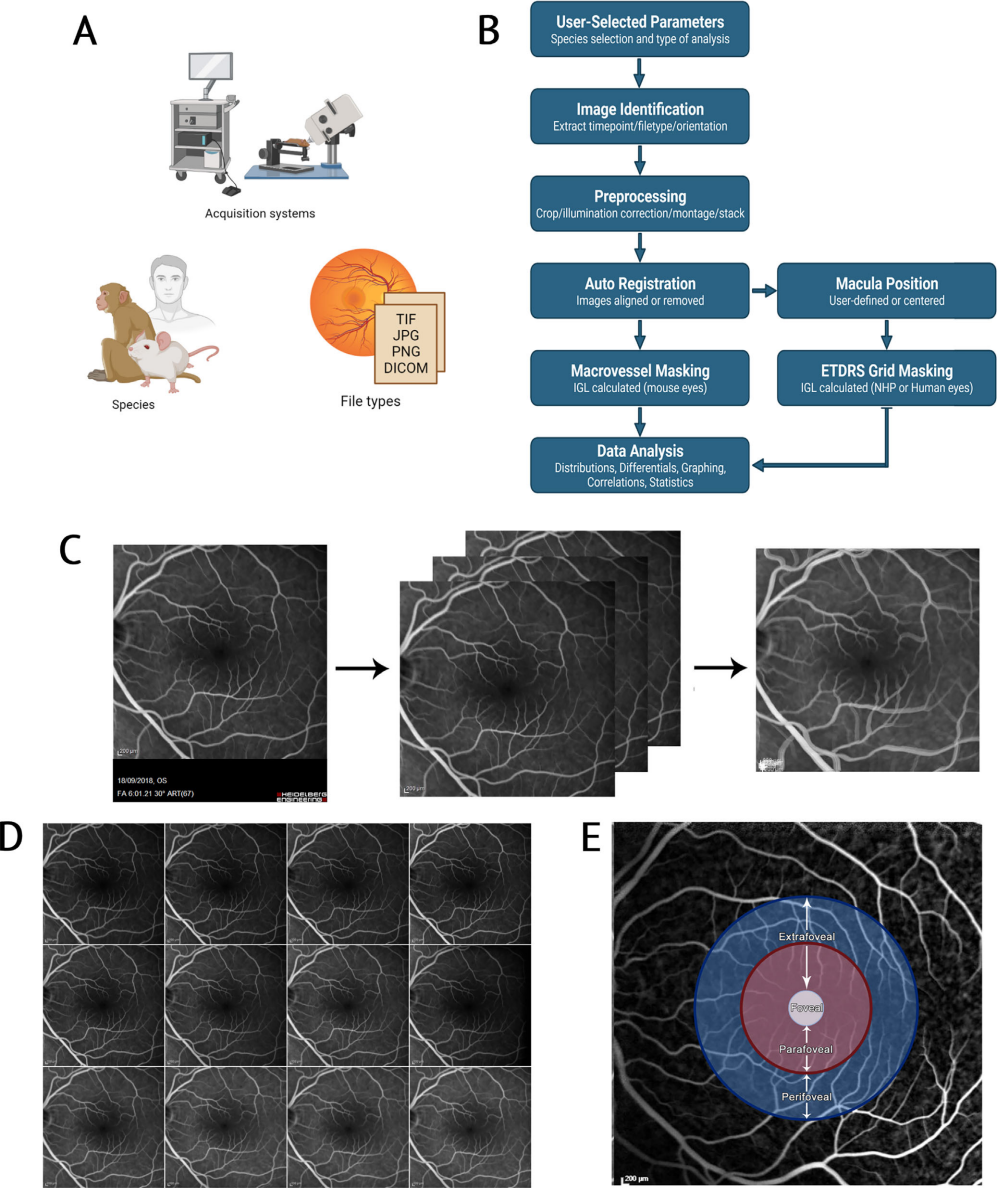
**FOVAS software was developed to automate high-volume FFA processing, facilitated by a graphical user interface to control several parameters for analysis.** (**A**) FOVAS has been designed to accommodate a range of acquisition hardware systems, species, and file types. (**B**) Schematic of the processing workflow. (**C**) Standard preprocessing workflow. Input images are cropped to exclude machine information, all images are then registered to account for eye movement during acquisition, and, finally, a MIP is generated. (**D**) Quality control images are generated, including a montage of all input images for each participant. (**E**) An ETDRS grid is applied to all registered images using the macula as its center point, defining the regions of interest.

The FOVAS user interface allows for easy selection of species and acquisition hardware prior to processing, registration, and analysis. Specifically, the software was designed to allow for a translational tool that would be applicable to numerous species: predominantly rodent, NHP, and human. Multiple hardware cameras and associated file formats are supported (Fig. 1A). Central to the analysis pipeline (Fig. 1B) is an automated registration tool that corrects for movement artifacts that will exist in all FFA analysis, such that a perfectly aligned series of FFA images will be created after initial preprocessing. A resulting MIP is created for downstream data processing (Fig. 1C). Quality control images are additionally generated as part of the processing steps, including an image montage, to easily review processed image data and monitor subsequent processing steps (Fig. 1D). Finally, in the context of the primate (human and NHP), FOVAS allows for segmentation of the retinal fundus image via the superimposition of an ETDRS grid such that downstream analysis can isolate various macular regions (Fig. 1E). To assess the circadian nature of iBRB permeability, we applied FOVAS to images generated in the morning and evening in the mouse, NHP, and human datasets.

### Young Adult Mice Display Significant Diurnal Variations in Fluorescein Signal

**Figure 2.**
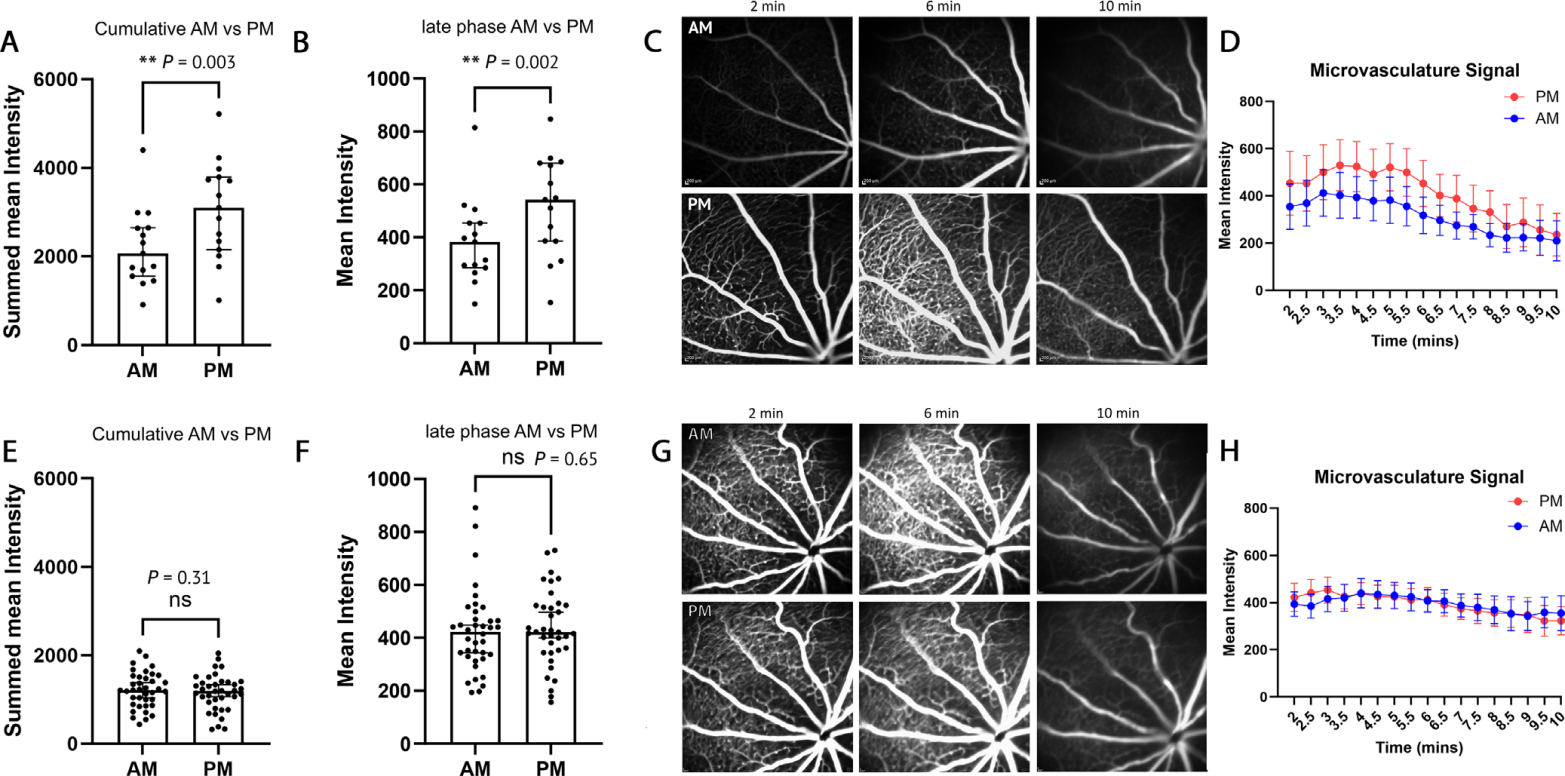
**FOVAS validation and assessment of morning versus evening FFA signal in young adult mice.** (**A**, **B**) Assessment of <12 month-old-wild-type mice in the morning and evening. The percentage change of fluorescein in the evening was calculated for the cumulative signal across all images in the imaging session (**A**) and the late phase after fluorescein injection (**B**). A significant increase in intensity was observed in the evening within the microvasculature at the late phase time range. (**C**) Representative images from in the morning (*top*) and in the evening (*bottom*). (**D**) Plot of average fluorescein intensity over time for both morning (*blue*) and evening (*red*) across all mice. (**E**, **F**) Assessment of >18-month-old wild-type mice in the morning and evening, showing the cumulative signal across the 10 minute imaging session (**E**) and the pixel intensity of the late phase (**F**). (**G**) Representative images from in the morning (*top*) and in the evening (*bottom*) taken at selected time points. (**H**) Plot of average fluorescein intensity over time for both morning (*blue*) and evening (*red*) across all mice.

Functionality to process images from mice were incorporated into the FOVAS software. The microvasculature was isolated from major vessels, and intensity was measured on this singular region. Because an ETDRS grid cannot be placed on mice to ensure longitudinal consistency in the analyzed retinal area, the field of view (FOV) of the entire image was taken for analysis. Sub-setting images for the common FOV across images could be more appropriate in the future; however, using the full FOV does not negatively impact the results observed (*n* = 11) (Supplementary Figs. S2A–S2C). Images were taken in both the morning and evening to assess baseline signal intensities in these naïve mice. The differences in microvasculature fluorescein signal between the morning and evening images were similarly shown in young (3–12 months old) wild-type C57 mice. The cumulative signal across the 10-minute exposure time frame showed that there was a significant increase in intensity in evening compared to morning images of 31.7% (*n* = 15, *P* = 0.003) (Fig. 2A). Examining the late phase specifically, there was a significant increase in signal in the evening (26% difference, *P* = 0.002) (Fig. 2B). Representative frames are shown at 2, 6, and 10 minutes from both morning and evening groups (Fig. 2C). In all animals, fluorescein intensity over time (Fig. 2D) clearly showed a decrease in signal 2 minutes after injection in the morning (blue), but the signal persisted for longer in the evening (red).

Interestingly, this difference was not seen in older mice greater than 18 months (ranging from 18 to 24 months of age). Looking first at the cumulative signal, there was no significant change in intensity in the evening images (–7%, *n* = 38, *P* = 0.31) (Fig. 2E). Similarly, at the late phase, there was a non-significant difference of 3% in signal between morning and evening (*P* = 0.65) (Fig. 2F). Representative frames are shown at 2, 6, and 10 minutes from both morning and evening groups (Fig. 2G). Intensity over time was plotted for all frames from all animals to demonstrate the average fluorescein intensity over the course of the experiment (Fig. 2H). All statistics shown in Figure 2 were obtained using a non-parametric Wilcoxon matched-pairs, two-tailed, signed-rank test.

### NHPs Display Significant Diurnal Variations in Fluorescein Signal

**Figure 3.**
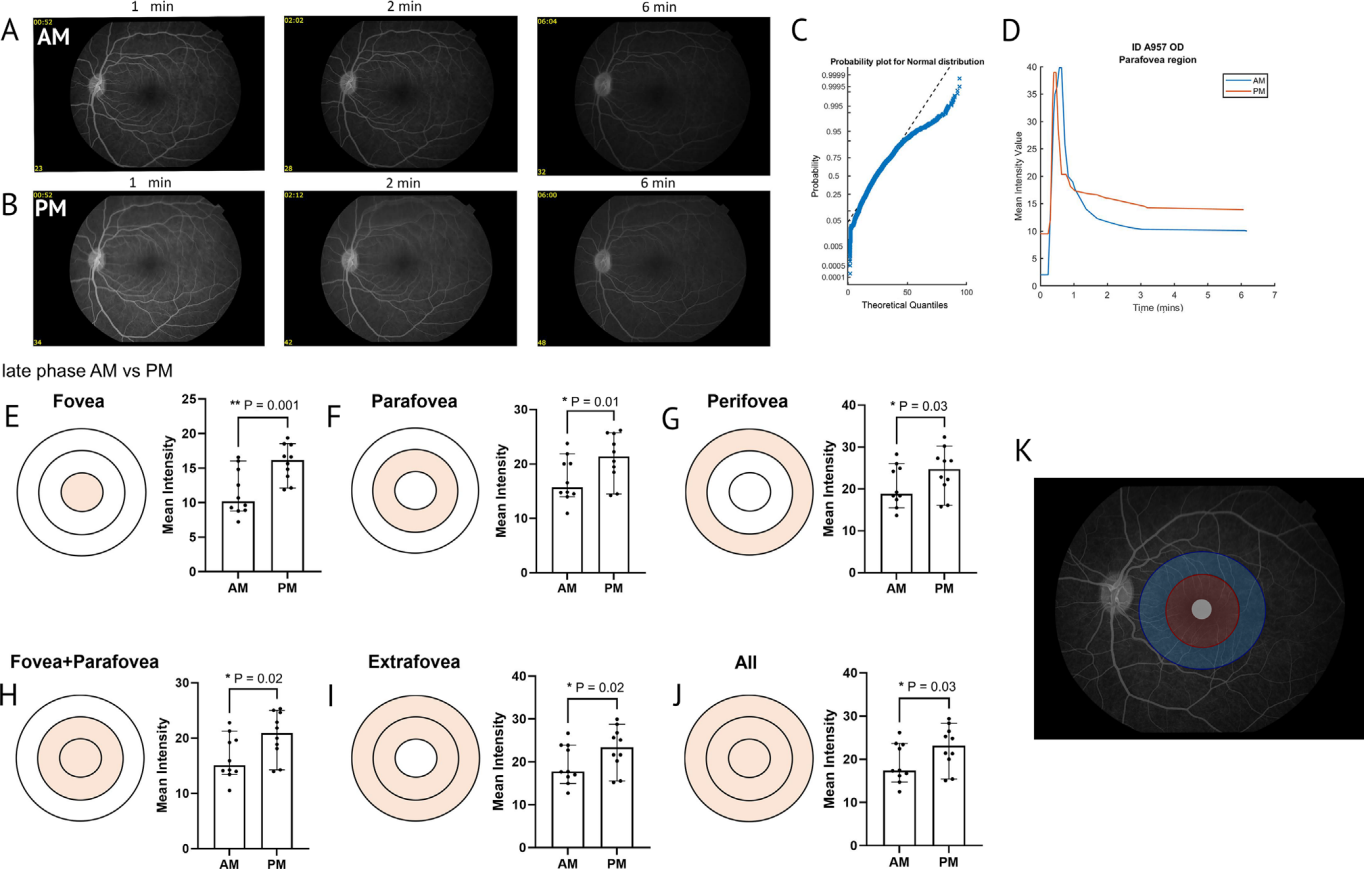
**FOVAS validation and assessment of morning versus evening FFA signal in NHPs.** (**A**, **B**) Representative images of NHP FFAs taken at approximately 1, 2, and 6 minutes after injection of fluorescein for both morning (**A**) and evening (**B**) time points. (**C**) The generated probability plot describes these data following a non-normal distribution, determining the downstream statistical methods used. (**D**) Representative plot from one animal describing the change in fluorescein intensity over time at both timepoints. (**E**–**J**) Fluorescein intensity at the late phase after injection was plotted for each region of the ETDRS grid to show the difference between morning and evening. These results indicate vessel leakage at this later time range. (**K**) A representative image of an FFA with the overlayed ETDRS grid defining the regions of interest.

To explore the translation of this circadian differential and to validate the software in larger animal species, we first looked at an untreated young adult NHP dataset. Our findings corroborated the results previously described,^17^ showing a similar percentage difference (within 10%) between morning and evening at both 2 and 6 minutes after fluorescein injection, validating our automated approach. FOVAS successfully determined the intensity profile of 10 healthy NHP eyes and compared the signal manifested in the morning compared to the evening in these macular eyes (Figs. 3A and 3B, respectively).

Initially, the generated data were automatically tested for normality using an Anderson–Darling test and plotted on a probability plot to assess whether the data followed a normal distribution. Because the data were non-normally distributed and both eyes from each animal were used, a mixed-effects statistical model was used (Fig. 3C). As part of FOVAS image processing procedures, signal intensity profile (i.e., fluorescein brightness) is plotted versus time after injection for each case, morning and evening, in this study. On first observation, the traces for all NHPs appeared to have a similar peak around 30 seconds after injection, and then showed a gradual decline, with a plateau beginning at around 4 minutes after injection, in agreement with the typical fluorescein cycle described in the literature. Beyond these time points, however, the evening signals appeared to plateau at a consistently higher mean intensity when compared to the morning signals (Fig. 3D). Shown in  Figure 3D is a representative animal from the larger group, the distribution for which is shown in Supplementary Figure S1A. The Anderson–Darling normality test rejected the null hypothesis, suggesting that these data followed a non-normal distribution (*P* < 0.0005).

In order to investigate this further, temporal analysis of the data was next performed to isolate time points within the late-phase post-fluorescein injection to examine the plateaued signal. It became apparent that the differences between morning and evening manifested in these later stages of fluorescein circulation, shown in Figures 3E to 3J (41% difference, *P* = 0.001 for fovea region; 29% difference, *P* = 0.01 for parafovea; 17% difference, *P* = 0.03 for perifovea; 30% difference, *P* = 0.02 for fovea plus parafovea combined; 21% difference, *P* = 0.02 for extrafovea; 22% difference, *P* = 0.03 for all regions). This indicates that, in the evening, fluorescein clearance is reduced, likely because it has “leaked” out of the vessels and is not being properly circulated. This is in contrast to morning, where a tighter barrier in the vasculature prevents fluorescein leakage, allowing it to clear from the retina faster. An example of the placement and boundaries of the ETDRS grid layers is provided in Figure 3K.

### Young Adult Healthy Eyes Display Diurnal Variations in Fluorescein Signal in the Central Macula That Is Lost With Age

**Figure 4.**
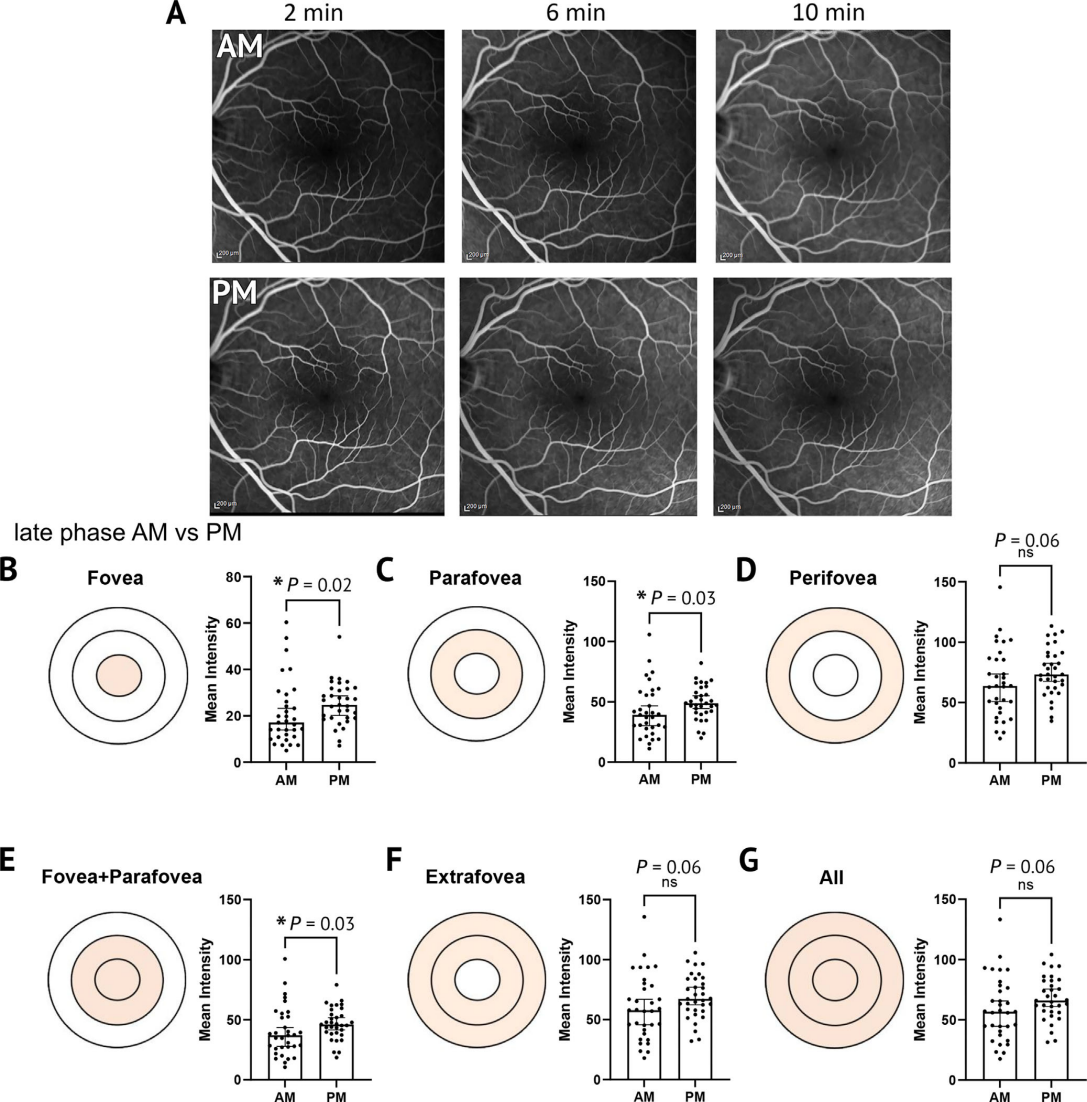
**FOVAS validation and assessment of morning versus evening FFA signal in young adult healthy human subjects.** (**A**) Representative images for these participants are displayed for both morning and evening imaging sessions. (**B**–**G**) The fluorescein intensity in the morning and evening is shown for images taken from the late phase of fluorescein injection. The associated diagrams indicate the region within the ETDRS grid currently being assessed. A significant increase in intensity was observed in the evening at this time range for the fovea, parafovea, and fovea plus parafovea regions.

To further validate the software in human eyes, we sought to examine the morning/evening signal differential in an expanded cohort of young adult healthy retina participants.^17^ In the young adult healthy retina, the late phase time range showed consistent differences between the average morning and evening fluorescein signal with a mean percentage difference of 25% for the foveal region (*P* = 0.02), 16% difference for the parafovea (*P* = 0.03), 16% difference for the perifovea (*P* = 0.055), 16% difference for the fovea and parafovea combined (*P* = 0.03), 15% difference for extrafovea (*P* = 0.06), and 15% difference for all regions averaged together (*P* = 0.055, *n* = 32) (Figs. 4A–G). The distribution of all data at the parafovea is shown in Supplementary Figure S1B. The Anderson–Darling normality test rejected the null hypothesis, suggesting that these data followed a non-normal distribution (*P* < 0.0005). All statistics for Figure 4 were obtained using a non-parametric Wilcoxon matched-pairs, two-tailed, signed-rank test. These data not only agree with the previously analyzed data but also highlight the contributions of each of the ETDRS regions to the observed fluorescein changes, with the central macular regions showing the most significant circadian differences. This suggests that vessel leakage in the evening is most prominent in the inner macula.

**Figure 5.**
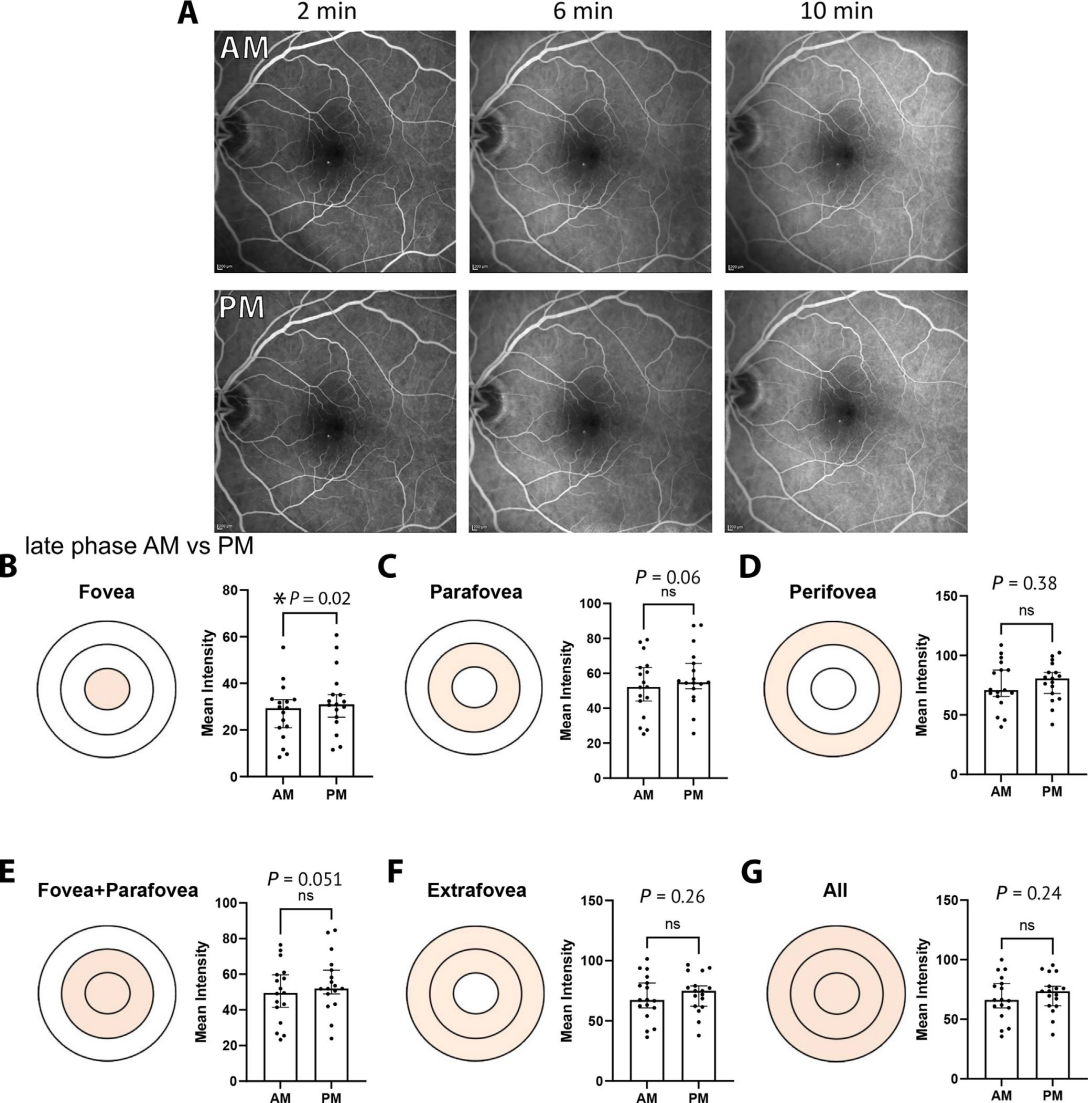
**FOVAS assessment of morning versus evening FFA signal in older healthy human subjects.** (**A**) Representative images at select time points for these participants in both morning and evening imaging sessions. (**B**–**G**) The fluorescein intensity in the morning and evening within the late phase after fluorescein injection. The associated diagrams indicate the region within the ETDRS grid currently being assessed. A significant increase in intensity in the evening was observed only in the fovea.

We then repeated this study in a new cohort of older healthy retina participants (65 years and older) to observe if the circadian pattern held (Figs. 5A–G). Intriguingly, this signal change was lost in the parafovea of the older cohort but remained at the fovea (Fig. 5B). For the late phase time range specifically, a mean percentage difference of 19% for the foveal region (*P* = 0.049, *n* = 17) was the only significant differential signal in the evening compared to morning. The distribution of all data at the parafovea is shown in Supplementary Figure S1C. The Anderson–Darling normality test rejected the null hypothesis, suggesting that these data followed a non-normal distribution (*P* < 0.0005). All statistics for Figure 5 were obtained using a non-parametric Wilcoxon matched-pairs, two-tailed, signed-rank test.

**Figure 6.**
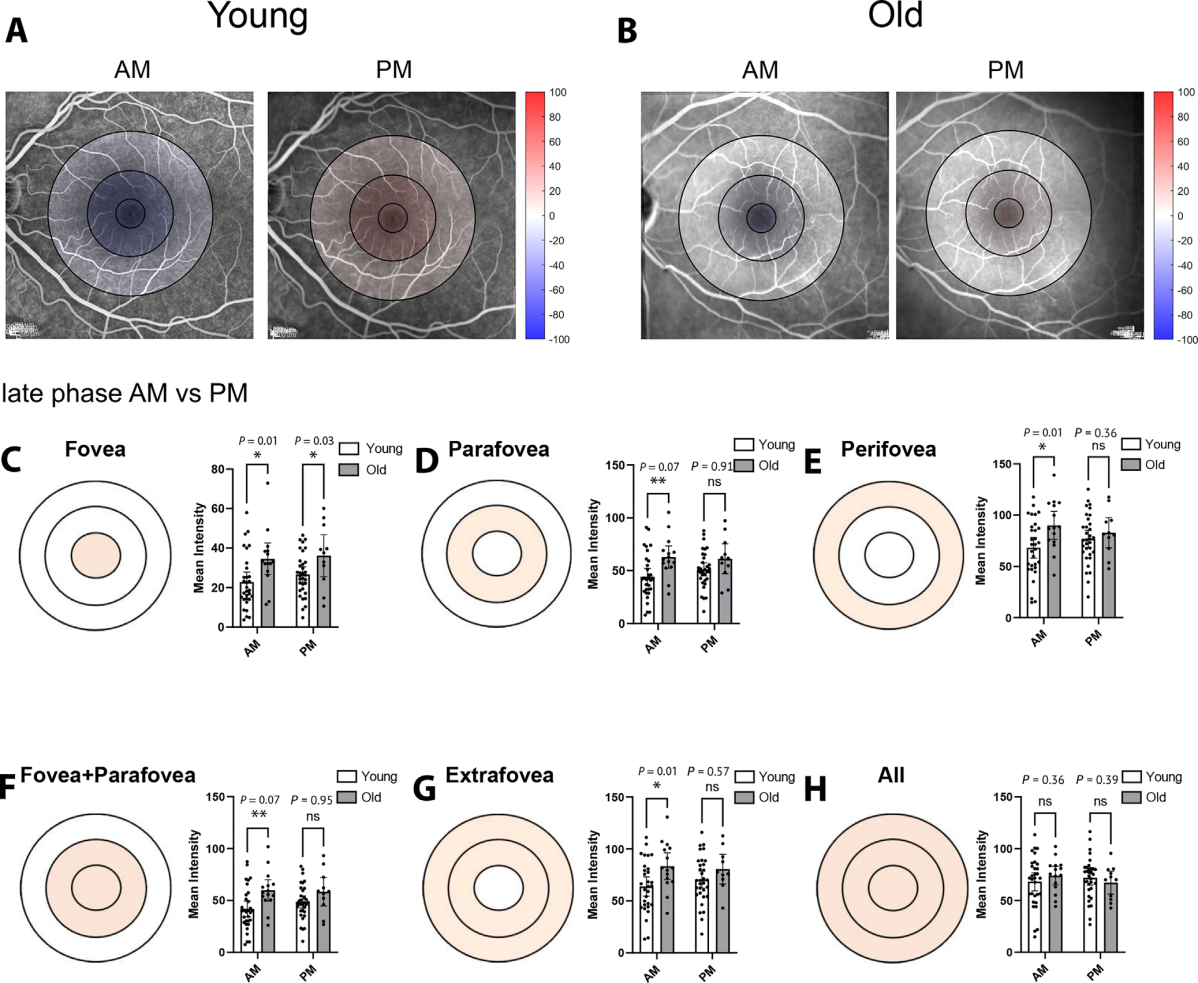
Age-related changes to the circadian dynamics of vessel integrity in human eyes. (**A**, **B**) Shown are representative circadian vessel integrity color maps for the young adult (**A**) and older adult (**B**) human cohorts. These maps indicate the percentage change in fluorescein signal for each participant between evening and morning at each ETDRS region, with progressively *darker red* *coloring* indicating greater vessel leakage in the evening. (**C**–**H**) Fluorescein intensity in the morning and evening was compared across the young adult and older adult human cohorts in a two-way analysis for each ETDRS region.

Circadian vessel leakage color maps were generated to indicate the regional changes in the inner retinal vasculature in the evening versus morning to summarize the above findings (Figs. 6A, 6B). Darker red colors indicate a greater percentage change in fluorescein signal, as seen in the representative image for the younger group in the evening (Fig. 6A). The lack of coloring in the representative image from the older cohort indicates no change in the evening in the ETDRS regions (Fig. 6B). Two-way mixed-effects analysis with multiple comparisons was performed on both the young adult and older adult groups, comparing age group and time point (morning vs. evening) to identify where the differences between the ages lay. Similar trends existed within the late phase; however, looking at single, user-specified time points (6 minutes specifically), it was observed that, in all segmented regions of the ETDRS grid, the fluorescein signal was greater in the morning when comparing the older versus younger participants (Figs. 6C–H). There was only a significant difference between the cohorts in the evening at the fovea (*P* = 0.03) (Fig. 6C), indicating that age-related changes in circadian vessel integrity homeostasis occur most substantially in the morning.

## Discussion

It is now well accepted that microvascular dysfunction is commonly associated with the development of numerous neurological disorders.^21^ Additionally, BBB disruption has been shown to be compromised in the aging brain and likely predisposes to cognitive decline and ultimately dementia.^22^ As an extension of the CNS, the retina is essentially a light-sensitive portion of the brain, and, in that regard, the retinal vasculature and iBRB have almost identical properties compared with the BBB, limiting the extravasation of bloodborne materials and maintaining very low rates of fluid phase transcytosis.

Our novel approach to automated FFA quantification provides several readouts for vessel permeability, a growing field of study that implicates vessel function and permeability in a range of ocular disorders. This permeability is described by FOVAS using several analytical metrics including temporal and cumulative fluorescent signal at anatomically aligned retinal sectors. Analyzing these different phases enhances the ability to distinguish changes in different retinal layers, with early-phase measurements primarily reflecting choroidal permeability and later-phase measurements capturing signals enriched from the inner retinal vasculature.^17^ Although other tools exist that also generate color-coded retinal maps and provide quantitative metric outputs, none to date describes leakage of the inner retinal vessels from multiple regions of the macula.

Our data highlight the potential of FOVAS as an analytical tool for FFA, and we propose that this software package will prove to be beneficial for both researchers and clinicians to study retinal diseases in preclinical, clinical, and drug development environments. Compatibility with multiple hardware acquisition systems, species, and file types allows standardizing quantification for a range of FFA analyses. Additionally, the continued growth of the above datasets will allow them to serve as reference standards when comparing new disease groups or individual participants.

There remain significant challenges quantifying FFA images among datasets. One such challenge is that FFA images are typically acquired using an adjustment of acquisition gain settings to attain an optimal image. We have tested a range of settings across instruments and have ensured that all participants in our study are imaged using the same instrument and acquisition settings for comparison. Choice of anesthesia and dose in the case of animal studies is an important factor for vascular hemodynamics and must also be kept constant. Additionally, a washout period must be sufficient to clear any existing fluorescein. We found that it was necessary to perform morning and evening measurements on separate days to allow enough time for fluorescein clearance between procedures. In addition, we also alternated whether morning or evening measurements were performed first. Other factors such as volume, concentration, and rate of fluorescein injection were kept constant among participants. We ensured that patient positioning, exported image resolution, and image dimensions were kept consistent across participants to control image scale. Axial length was not controlled in this study due to the absence of optical biometry data, a potential limitation of this study, but it could be implemented into FOVAS in future versions. Subjective exclusion of poor-quality images prior to automated analysis remains a limitation of the software; however, automated checks on quantitative metrics including brightness, sharpness, and contrast are already being developed for integration into FOVAS. In addition to registration of images taken within a FFA session, registration across imaging sessions could prove to increase the accuracy and robustness of longitudinal studies and will be a useful addition to future versions of the software. Optical coherence tomography angiography (OCTA) presents an attractive non-invasive method for retinal vessel imaging without the need for injecting dye. However, without injecting a dye there is no leakage indicator, hence size-selective iBRB permeability is not easily detected with OCTA. As such, FFA remains a useful tool for assessing vessel leakage and other abnormalities, such as in uveitis, where multimodal imaging is considered the preferred approach.^23^

Our FOVAS software is positioned as a robust automated quantification tool for FFA images, validated against existing datasets. Through our validations, not only have we corroborated results previously described, but, with the added detail of regionality and temporal analysis of these images, we can also more accurately identify the exact retinal regions and fluorescein phases that contribute to vessel permeability changes. This capability has significant potential to facilitate studies where regional and temporal vascular changes have not yet been systematically examined. Moreover, the cohorts described in this manuscript provide valuable reference datasets for vessel permeability across age groups and times of day, which will support future investigations into disease cohorts.

In this study, we identified the inner macula, particularly the fovea and parafovea, as a major site of circadian variation in signal, with higher vessel permeability observed in the evening compared to the morning in young, healthy adult subjects. With age, this circadian differential appears to reduce, and to a greater extent in the parafovea. The data suggest that the elevated morning signal in older participants is responsible for the diminished circadian effect. This in turn suggests that, with age, the retinal vasculature does not “tighten” up again in the morning, leaving the vessels outside the fovea in a permanently “open” state. These biological insights are not obvious or easily demonstrated without the quantification provided by FOVAS. The corresponding datasets have been incorporated into FOVAS as reference standards to enhance reliability and support its application to healthy and diseased cohorts in future studies.

Persistent vessel leakage can contribute to a variety of chronic ocular disorders typically seen in the aging retina, including macular edema, ischemia, or fibrosis. With regard to treatment strategies aimed at regulating modulators of the iBRB, it must be noted that there are now multiple avenues of investigation with the end goal of regulating tight junction proteins such as claudin-5 to stabilize the retinal vasculature and therefore stabilize disease progression. Using small molecules such as RepSox^24^ clearly shows that pathology can be attenuated; however, more advanced methods such as adeno-associated virus (AAV)-based delivery of claudin-5 could also prove efficacious. More indirect methods aimed at tapping into signaling networks that will result in claudin-5 upregulation are also on the horizon, and how these agents work in tandem with anti-vascular endothelial growth factor (VEGF)^25^ or anti–complement-based drugs will be revealing.^26^

## Conclusions

In summary, this study introduces and validates FOVAS (Fluorescent Ocular Vasculature Analysis Suite), an automated, cross-species platform for the objective quantification of Fundus Fluorescein Angiography (FFA) images. Utilising FOVAS, we successfully demonstrated a significant circadian rhythm in inner blood-retina barrier (iBRB) integrity, characterized by higher vessel permeability (greater fluorescein signal) in the evening (PM) compared to the morning (AM) in young adult mice, non-human primates (NHPs), and humans. Critically, this diurnal difference was found to be significantly attenuated with age across all species, particularly in the parafoveal region of older human participants, suggesting an age-related loss of morning “tightening” of the retinal vasculature. These findings underscore the importance of considering the time of day in both clinical diagnosis and pre-clinical research and provide robust reference datasets for future investigations into retinal vascular disorders. FOVAS, with its regional and temporal analytical capabilities, is positioned to be a vital tool for advancing our understanding of iBRB dynamics in health, aging, and disease.

## Supplementary Material

Supplement 1
