# Supplementary material for: Automated Assessment of Retinal Vascular Integrity Across Species
Source: Invest Ophthalmol Vis Sci. 2025 Dec 15;66(15):46. doi: 10.1167/iovs.66.15.46 (PMC12710776; doi:10.1167/iovs.66.15.46)
Supplement: Supplement 1 [file iovs-66-15-46_s001.pdf]

Figure S1

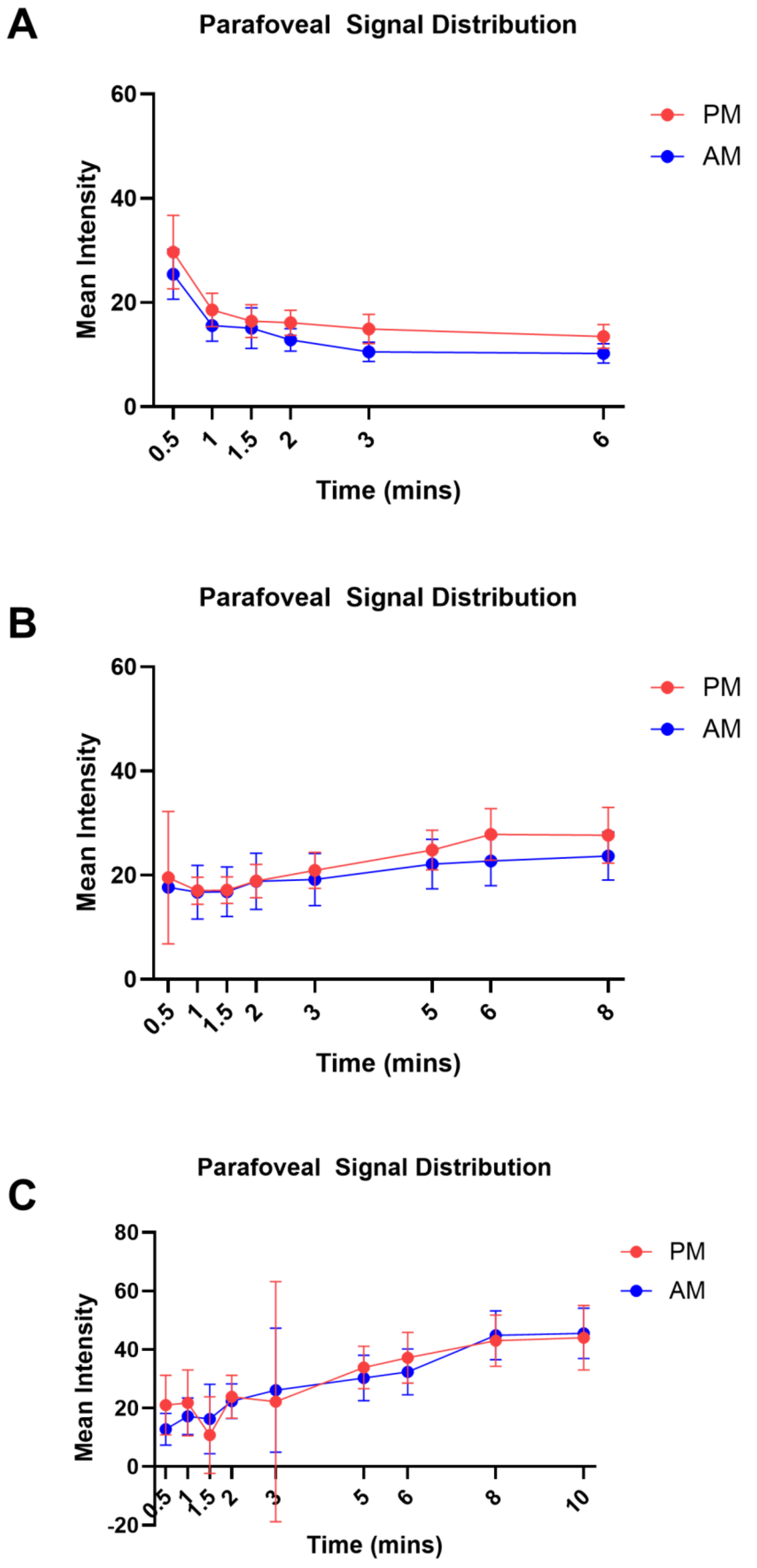
A
Parafoveal Signal Distribution
Mean Intensity
Time (mins)
PM
AM
B
Parafoveal Signal Distribution
Mean Intensity
Time (mins)
PM
AM
C
Parafoveal Signal Distribution
Mean Intensity
Time (mins)
PM
AM

Figure S1. Data distributions at the parafovea across species
The distributions shown are plotted using the data from the parafovea region across (A) NHPs, (B) young adult participants, and (C) aged participants respectively. The data is comprised of all subjects in each group at the given timepoints for that region.

Figure S2

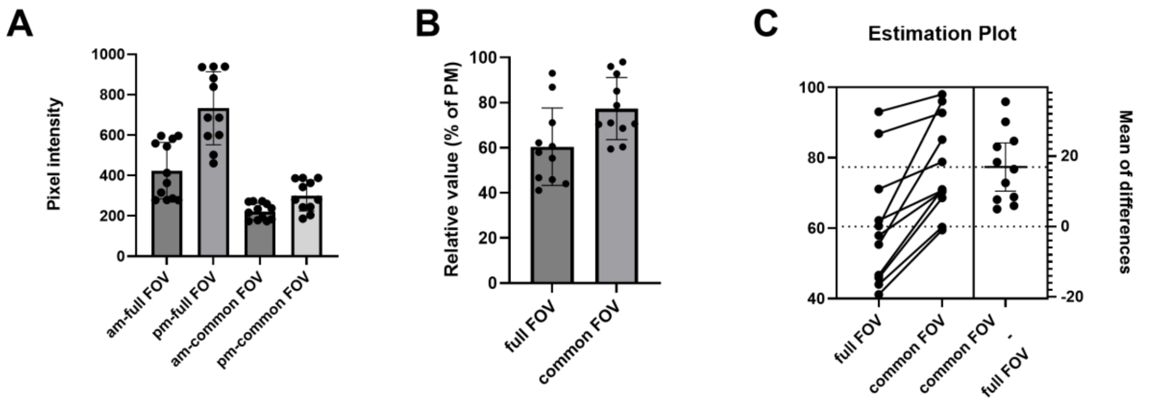

Figure S2. Field of view assessment in murine eyes
Comparison of pixel intensity values when analysing the entire field of view (FOV) of an image, verses only analysing the common FOV area across sessions in murine images (A). There is an evident increase in the intensity of PM images in both analysis cases, however they show a similarly elevated difference (B). The magnitude of effect is greater in the common FOV area.
